# Assessing the Financial Value of Decentralized Clinical Trials

**DOI:** 10.1007/s43441-022-00454-5

**Published:** 2022-09-14

**Authors:** Joseph A. DiMasi, Zachary Smith, Ingrid Oakley-Girvan, Andrew Mackinnon, Mary Costello, Pamela Tenaerts, Kenneth A. Getz

**Affiliations:** 1 Tufts Center for the Study of Drug Development, Tufts University, 145 Harrison Avenue, Boston, MA 02111 USA; 2 Medable, Inc, Palo Alto, CA USA

**Keywords:** Decentralized clinical trials, Clinical development phases, R&D costs, Pharmaceutical sales, Expected net present value, Return on investment

## Abstract

**Background:**

Deployment of remote and virtual clinical trial methods and technologies, referred to collectively as decentralized clinical trials (DCTs), represents a profound shift in clinical trial practice. To our knowledge, a comprehensive assessment of the financial net benefits of DCTs has not been conducted.

**Methods:**

We developed an expected net present value (eNPV) model of the cash flows for new drug development and commercialization to assess the financial impact of DCTs. The measure of DCT value is the increment in eNPV that occurs, on average, when DCT methods are employed in comparison to when they are not. The model is populated with parameter values taken from published studies, Tufts CSDD benchmark data, and Medable Inc. data on DCT projects. We also calculated the return on investment (ROI) in DCTs as the ratio of the increment in eNPV to the DCT implementation cost.

**Results:**

We found substantial value from employing DCT methods in phase II and phase III trials. If we assume that DCT methods are applied to both phase II and phase III trials the increase in value is $20 million per drug that enters phase II, with a seven-fold ROI.

**Conclusions:**

DCTs can provide substantial extra value to sponsors developing new drugs, with high returns to investment in these technologies. Future research on this topic should focus on expanding the data to larger datasets and on additional aspects of clinical trial operations not currently measured.

**Supplementary Information:**

The online version contains supplementary material available at 10.1007/s43441-022-00454-5.

## Introduction

The use of remote and virtual approaches and technologies to collect study data and improve participation access and convenience represents a profound shift in clinical trial execution. Although the deployment of many of these solutions—in combination referred to commonly as decentralized clinical trials (DCTs)—began a decade ago, the COVID-19 pandemic has facilitated rapid adoption given the necessity to reduce the risk of transmitting infection and minimize delays and disruptions [1].

Guidance for ongoing trials during the COVID pandemic from the Food and Drug Administration and the European Medicines Agency have encouraged research sponsors to support both hybrid executional approaches (i.e., use of select technologies and solutions to reduce the number of in-person visits at clinical research sites) and fully virtual approaches (i.e., use of mobile technologies, telehealth, in-home visits, drugs and devices delivered to the patient, and no in-person site visits) [2, 3].

The promise of DCT methods is compelling and may address a number of challenges that have long plagued drug development. By offering the opportunity to participate in clinical trials remotely and virtually, for example, DCT solutions may improve patient access most notably to minority and under-served patient communities from harder-to-reach geographic areas. DCTs may also reduce the burden of participation as select clinical research data can be collected in the background, in the comfort of a patient’s home or during a patient’s normal daily routine. As such, DCTs hold the potential to improve patient adherence to the protocol and may increase overall retention rates [4].

DCTs promise to leverage clinical research data faster and more effectively. The volume, frequency and variety of data collected—from electronic case report forms, patient health records, from sensors and wearable devices and diagnostics—may offer the opportunity to interrogate and draw insights from the data sooner, reduce the number of patients required, and may increase statistical power [5].

The deployment of DCTs may also offer operational efficiencies through the automation of certain manual data collection tasks, more frequent and convenient communication with study volunteers, and more focused and productive investigative site personnel [1, 6, 7].

Case examples and anecdotal reports in peer-review, trade press and in marketing communications indicate that the promise of DCTs is being realized. A growing number of sponsors, contract research organizations (CROs), DCT software and technology providers have shared their experiences with, and the positive impact of, DCT deployments [5, 8, 9]. Many sponsor companies have established internal mechanisms—such as dedicated teams and functions—to assess and pilot DCT activity [5]. With growing implementation experience, some have also noted challenges introduced by DCTs including adapting to new clinical trial operational work flows and the upfront and ongoing cost of training and providing technical support to investigative site personnel and study volunteers [10].

To assist research sponsors in evaluating whether to deploy DCTs, the Tufts Center for the Study of Drug Development (Tufts CSDD) an independent academic group within the Tufts University School of Medicine—in collaboration with Medable Inc., a technology provider of DCT software as a service (SaaS)—conducted a study quantifying the net financial impact of deploying remote and virtual approaches and technologies to support clinical trial execution. The definition of a DCT applied here is that described by *The Clinical Trials Transformation Initiative* (CTTI) and is provided in the Supplemental Data File. A description of the Medable technology is also provided.

This paper presents our application of the expected net present value (eNPV) method using benchmark and proprietary data on actual experiences. The eNPV approach is a commonly used, and widely recognized, risk-adjusted financial modeling technique. To our knowledge, such an assessment on DCT usage has not been performed previously.

It is our hope that the results of this assessment will help inform R&D sponsors in deciding whether to invest in, and in determining the amount of resources to allocate, to support the deployment of DCT solutions.

## Data and Methods

Benchmark protocol performance data for trials with and without DCT elements were calculated from a dataset of both large and small molecules collected during previous research conducted by CSDD [11]. In this previous research, protocol complexity data were provided on phase I-III protocols by 20 sponsor and CRO companies for trials conducted both in and outside the United States. To be included in the dataset, protocols had to have been finalized between January 2013 and December 2018 with a primary clinical trial completion date or database lock date before December 31st, 2019. In total, data on 220 protocols were included. Data on DCTs for phase I trials in our datasets is limited, so we focus in this analysis on phase II and phase III trials. The CSDD benchmark dataset contains information on 160 phase II and phase III protocols.

Individual values 3 standard deviations away from the mean for any particular variable were considered outliers and were removed from the dataset. All other data for the affected protocols were retained.

To calculate benchmarks for performance variables for trials with and without DCT elements, protocols were divided by data sources used. For the previously mentioned study, companies indicated which protocols included data from devices and apps (such as wearable devices, the use of smartphones or tablet applications, or other sensors such as glucose monitors, smart pills, or ambient sensors), which protocols included the use of real-world evidence, and which protocols included data from electronic health records (data collected from electronic health record systems). Any protocol that reported including data from devices and apps (e.g., electronic clinical outcome assessments [eCOAs], diaries, and connected sensors), real-world evidence, or electronic health records (or any combination of the three) was classified as having DCT elements. Protocols that did not indicate the use of data from these sources was classified as not having DCT elements. Nearly 90% of the protocols classified as having DCT elements for our analysis involved devices and apps.

Means, medians, and counts were calculated for several variables measuring trial performance including total trial cost, number of patients screened, number of patients enrolled, screen failure rate, dropout rate, and time from protocol approval to clinical report. These benchmarks were calculated by DCT classification and phase.

Protocol data were stored as an excel file on a secure, shared, online drive, and analysis was conducted in SAS 9.4. For more information on collection of these benchmark data, please refer to Tufts CSDD’s previous publication [11].

In addition to the Tufts CSDD benchmark data for trials with and without DCT elements, we utilized a sample of data from Medable contracts to determine average implementation costs for 33 and 26 multi-specialty phase II and phase III clinical trials, respectively, with DCT elements for trials in any location. For this analysis, we focused on phase II and III because DCT utilization for phase I trials has been limited to date.

### Expected Net Present Value Model

To quantify the value of adopting DCTs we employed a methodology that has been widely accepted and practiced across industries for evaluating the value of investment project portfolios. The eNPV method accounts for R&D investment cash flows, risks in reaching the marketplace, costs of commercialization, and projected sales. Cash flows from different periods are made comparable through discounting. For industrial investment projects that discounting is achieved through the application of a company cost of capital.

The major levers impacting eNPV for drugs in development include clinical phase durations, the likelihood of proceeding from one development phase to the next phase, development phase costs, effective patent lifetimes, and shifts in the shapes or levels of sales curves. We can measure the value and financial viability of a change in any or all of these levers with the eNPV model. Putting aside strategic and other intangible concerns that may affect decision-making, a change in the drug development paradigm is financially better than the status quo if the increment in eNPV from adopting the change relative to the status quo is positive.

The method has been applied recently to a number of hypothesized improvements to the drug development process, including adopting patient engagement methods [12], integrated formulation development, real-time manufacturing and clinical testing [13], and single-source versus multi-vendor outsourced biopharmaceutical manufacturing [14]. We examine here another departure from the standard drug development paradigm and assess the value of DCTs to drug sponsors by determining the increment in eNPV from industry adopting virtual and remote approaches and technologies across its portfolio of investigational drugs for either phase II or phase III trials, or for both phases.

### Development Parameters

Values used for the key elements of the base case (i.e., status quo) model are shown in the Supplemental Data File (Tables S1 and S2). The development time, development risk, and development cost parameter values are taken from a Tufts CSDD study of pharmaceutical R&D costs [15]. We also use the weighted average company cost of capital from that study (10.5%) and apply it to our cash flows. Non-R&D cost parameters (e.g., marketing, production, and administrative costs) are based on general industry standards.

The base case time from the start of phase II testing to marketing approval taken from the literature is 77 months (Table S1), while the assumed time from the start of phase III to marketing approval is 47 months (Table S2). We analyze the value of employing DCTs from the perspective of a drug sponsor at either the start of phase II testing or the start of phase III testing.

eNPV is a risk-adjusted discounted cash flow metric. Development risk is therefore central to the model. Since we start at either the beginning of phase II or phase III, the compounds are already de-risked to some degree and their value is greater than if we considered a portfolio of drugs that were starting at phase I or earlier. So, while the study we use for risk metrics has an approval success rate of 11.8% for drugs that enter clinical testing, the approval success rate is 19.9% if we consider drugs that have progressed to phase II testing and 56.0% if the set of drugs we consider have already progressed to phase III. Thus, the eNPV levels will be higher at the start of phase III than they are if we are considering drugs that are entering phase II.

The clinical phase costs used as base case parameters and shown in Tables S1 and S2 are costs per investigational drug entering the phase of interest. Thus, they are costs at the molecule level, not at the indication level. This fact, along with the risk data, is important for characterization of the results. The eNPV increments are changes in value from applying DCT methods across all trials for all indications pursued for drugs entering a given clinical phase. The costs are spread uniformly by month across the total duration of the given phase.

### Commercial Parameters

The base case sales curve is based on data we collected on actual annual sales histories and annual future sales projected by consensus analyst forecasts found in two commercial pipeline databases (*Cortellis* and *Adis Insight*). The sales histories and forecasts were obtained for new molecular entities (NMEs) and new biologic entities (NBEs) approved by the Food and Drug Administration (FDA) from 2007 to 2017. The cutoff of 2017 allows us to have significant histories of actual sales for the sample drugs.

For the sales data we determined peak annual sales and the number of years from launch to peak sales. The sales dataset contains sufficient sales information on 243 of 343 NMEs/NBEs approved by the FDA’s Center for Drug Evaluation and Research (CDER) from 2007 to 2017. Mean and median peak sales for these data are $1,852 million and $717 million in year 2020 dollars, respectively. The median time to peak sales is nine years. However, for some drugs sales were still increasing as of the latest year for which there were data. Thus, we make the reasonable assumption that median time to peak sales is 10 years. We do not increase the average peak sales estimate as we do not have information on higher sales for the drugs where sales were still increasing. Consequently, our eNPV value estimates are likely somewhat conservative.

We assume that sales during the exclusivity period follow a logarithmic growth curve. With this assumption, a peak sales estimate, and an assumed exclusivity period, we can determine sales levels up to the time when generic drugs or biosimilars start to erode patent-protected prescription drug sales. We assume a moderately aggressive decline in sales after the loss of exclusivity. The period of exclusivity is taken to be 11 years after regulatory marketing approval.^1^

This is consistent with studies in the literature on effective patent lifetimes (time from marketing approval to the loss of patent protection) [16–18]. We also assume a 25-year product lifecycle. Beyond that sales would be very low and discounting back to the start of a development phase would decrease even those small amounts significantly.

### Implementation Costs

The benefits of DCTs must be weighed against any additional costs. We take these costs of implementation as the average contract values in the Medable data by phase during the period 2020–2021. Under the assumption that DCT methods will be employed across all trials for a given phase, we multiply the average contract values by an assumed average number of trials for a phase. Our base assumptions are that there will be four phase II trials for drugs that enter phase II, and three phase III trials for drugs that enter phase III. However, for our sensitivity analyses we examine the results under different assumptions about the number of trials in a phase (one to six).

The implementation costs are integrated in the eNPV model the same way as are the average clinical phase costs noted above (spread across the given phase and discounted back to the start of the phase). Based on the data available, the average implementation cost is $473,000 for a phase II DCT trial and $1,042,000 for a phase III DCT trial. Applying the assumed average number of trials for each phase, yields implementation costs of $1,892,000 for phase II and $3,126,000 for phase III.

Aside from assessing the value of DCTs as increments in eNPV, we also report on a return on investment (ROI) metric defined as the increase in eNPV from applying DCT methods divided by the implementation cost. We estimate ROI conservatively by implicitly assuming that the implementation cost in the denominator of the ROI ratio is paid in full at the beginning of the phase in question. When determining the denominator of the ROI metric for the case in which we assume that DCTs are applied to both phase II and phase III trials for a portfolio of drugs that enter phase II, we must combine the different implementation costs by weighting them according to their likelihood of occurrence. As shown in Table S1, the likelihood that a drug that enters phase II will progress to phase III is 35.5%. Thus, for the phase II plus phase III DCT case reported below the implementation cost used for the ROI metric is $1,892,000 + (0.355 x $3,126,000), or $3,001,730.

## Results

While these do not exhaust the many ways in which DCT deployments can benefit clinical trial performance, we have identified three factors that can increase eNPV and for which we have data. They are clinical phase cycle times, decreases in screen failure rates, and decreases in the number of substantive protocol amendments (a version of the protocol prepared and approved to correct a significant error or to make a significant change in a document already submitted to a regulatory authority). Our benchmark data suggest that phase II and phase III durations can be reduced at least 10%, or 3 months, for each phase. We conservatively assume 10% reductions in the time from the start of one phase to the start of the next phase. The reductions in time bring revenues for drugs that make it to market closer to the start of phase testing, and so increase cash flows after discounting.

Tufts CSDD data and analysis suggests that screen failures account for 11% of trial costs [19]. The data also indicate that the average screen failure rate for trials without DCT elements is 31.5% for phase II and 29.9% for phase III. The number of trials with screen failure data on trials with DCT elements in the Medable dataset is much larger than the number in the CSDD protocol database. So, we used the Medable data for screen failure rates for DCTs and the CSDD data for non-DCTs. Data for the other factors were either not clearly defined identically or not present in the Medable data.

In the Medable dataset, average screen failure rates are 24.1% for phase II and 20.1% for phase III. Therefore, when comparing screen failure rates for trials with DCT elements versus those without, we find a screen failure rate reduction of 23.5% for phase II trials and a 32.8% reduction for phase III. As a result, we assume that adopting DCT methods reduces trial costs by 2.58% for phase II and 3.61% for phase III.

Prior Tufts CSDD research indicates that the cost of a substantial protocol amendment is $141,000 for phase II trials and $535,000 for phase III trials [20]. Tufts CSDD benchmark data indicate that the number of substantive protocol amendments, on average, for phase II trials is 3.3 for trials with no DCT elements and 2.4 for trials with DCT elements. Similarly, Tufts CSDD data indicate that the number of substantive protocol amendments, on average, for phase III trials is 3.4 for non-DCT trials and 3.2 for DCT trials. Given the reductions in the number of amendments, the cost per amendment, and the assumed number of trials, costs for the base analysis are reduced by $507,600 for phase II and $321,000 for phase III because of fewer protocol amendments.

We also examined the data by therapeutic class, and found that the therapeutic class distributions were roughly similar for the Medable and CSDD datasets. The results can be found in the Supplemental Data File.

### Phase II DCT Value

**Fig. 1:**
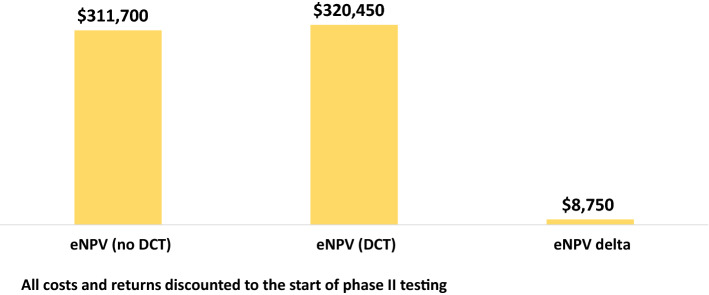
Increase in eNPV per Phase II Investigational Drug With Decentralized Clinical Trials (thousands 2020 USD)

Figure 1 shows our results for the adoption of DCT methods for phase II trials for a portfolio of drugs entering phase II. The analysis presumes that DCT methods are applied across all pre-approval phase II indications pursued. Without DCT elements, the eNPV, discounting to the start of phase II testing, is $311,700,000. Our model and data suggest that the value of introducing DCT methods to phase II trials increases eNPV to $320,450, 000. That is an increment in value of $8,750,000. The increment in value amounts to 2.8% of total estimated value for these drugs. The ROI for the base analysis is 4.62 (i.e., the return on investment is nearly five times the additional cost of implementing DCT elements).

Sensitivity analyses can be conducted for any of the parameters of the model. In Table 1, we examine the results for different assumptions about the extent to which phase II duration is reduced. The greater the reduction in cycle time, the higher the net financial benefit of DCTs. For our base analysis, we assumed a three-month reduction in phase II duration. If the reduction in cycle time is only one-month, the change in value is still positive and substantial. The return on investment is 1.61 times the investment cost. With a six-month reduction in cycle time, the increase in eNPV is $17 million and the ROI is 9.25.

**Table 1:** Increase in eNPV per investigational drug for phase II decentralized clinical trials (thousands 2020 USD): variation in assumed reduction in phase cycle time

Cycle time reduction (mos.)	eNPV delta	eNPV delta as percent of base eNPV (%)	ROI
1	$3042	1.0	1.61×
2	$5884	1.9	3.11×
3 (Base analysis)	$8750	2.8	4.62×
4	$11,641	3.7	6.15×
5	$14,588	4.7	7.69×
6	$17,499	5.6	9.25×

Costs and returns discounted to the start of phase II testing

*ROI* eNPV delta/Implementation cost

Results for several other single parameter sensitivity analyses are shown in the Supplemental Data File. We assumed that there are four phase II trials in the base analysis. That is a parameter that can be varied as shown in Figure S1. The results are not very sensitive to the assumed number of trials that are conducted in the phase. The calculated net benefit (delta eNPV) falls slightly with the number of trials. While the benefits from fewer protocol amendments increase with the assumed number of trials, the costs of implementation increase with the number of trials. On net, if the assumed number of trials is reduced from four to two, the value of DCTs increases by just 5.9% to $9,263,000. If the number of trials increases from four to six, then the change in eNPV decreases by 5.9% to $8,237,000.

We also examined changes in the implementation cost. It is thought that contract values for DCT projects might vary plus or minus 25% over the averages we have. We are interested here in averages, and not the extremes, but we can ask how the results would be affected if the average cost varied to this degree. Figure S2 shows that the results are even less sensitive to implementation cost than they are to the assumed number of trials. The change in eNPV for phase II DCTs varies only by 4.0% from the assumed value for the base analysis if the implementation cost is either 25% lower or 25% higher. In both cases the change in eNPV is positive.

Finally, we also examined different assumptions about the percentage reduction in screen failure rates from applying DCT methods (Figure S3). The base analysis screen failure rate differential for phase II trials is 7.4%. We calculated changes in eNPV if, instead, the screen failure rate differential was either 5% or 15%. Again, the results are not especially sensitive to this parameter. If the screen failure rate differential is only 5%, then the value of DCTs is 4.8% lower at $8,344,000. If the differential is 15%, then the delta in eNPV increases by 14.7% to $10,037,000.

We can also assess the relative importance of the three factors contributing to sponsor financial benefits by assuming that only one or two of the factors hold. Table 2 shows these results. If a reduction in protocol amendments is the only factor that applies (i.e., there is no reduction in phase II duration or decrease in screen failure rates), then the net benefits are negative, as the lower costs from fewer amendments are overshadowed by the cost of implementing the DCTs. If only a reduction in screen failure rates applies, then there is a net financial loss of 8%. The overall results are largely driven by the reduction in cycle time. If that is the only factor, then the return in terms of a change in eNPV is 3.76 times the investment cost.

**Table 2:** Increase in eNPV per investigational drug for phase II decentralized clinical trials (thousands 2020 USD): variation in combination of screen failure, amendments and cycle time effects

Factor effects	eNPV delta	eNPV delta as percent of base eNPV (%)	ROI
Amendments only	− $1014	− 0.3	-0.54x
Screen failure only	− $148	< − 0.1	-0.08x
Cycle time only	$7121	2.3	3.76x
Screen failure plus amendments	$224	< 0.1	0.12x
Amendments plus cycle time	$7497	2.4	3.96x
Screen failure plus cycle time	$8374	2.7	4.43x

Costs and returns discounted to the start of phase II testing

*ROI* eNPV delta/Implementation cost

### Phase III DCT Value

**Fig. 2:**
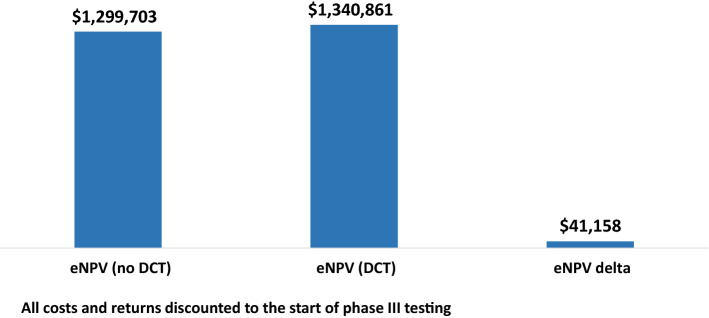
Increase in eNPV per Phase III Investigational Drug With Decentralized Clinical Trials (thousands 2020 USD)

We can analyze the value of DCTs for phase III trials in the same way we did for phase II. The perspective here, though, is that DCTs are applied to a portfolio of drugs that have entered phase III testing. Since we are starting from a point where the pipeline has been substantially de-risked because the drugs in the portfolio have already successfully transitioned from early-stage clinical testing, the base eNPV is much higher than it is for a portfolio of drugs that have only reached phase II. Figure 2 shows that the eNPV for non-DCT trials is $1,299,703,000, while applying DCT methods increases eNPV by $41,158,000. This increment in value amounts to 3.2% of the total value for the base case (no DCT). The ROI for the base analysis is 13.17, which means that the return is approximately 13 times the additional cost of implementing DCT elements.

For the base analysis we assume that phase III cycle time is reduced by three months when DCT methods are applied. As we did for phase II, we analyzed the increment in eNPV from DCTs for one to six months cycle time reductions (Table 3). Here, the increase in eNPV for DCTs for a one-month reduction in phase III duration ($17 million) is nearly twice that for phase II at a three-month reduction. The ROI for the phase III one-month reduction is 5.51, which is similar to that for a three-month reduction in phase II. At the high end of our sensitivity analysis, a six-month reduction in phase III cycle time results in an increase in value per investigational drug of $78 million and a nearly 25-fold return on investment. As with phase II, the relative importance of the three factors that contribute to sponsor financial benefits for phase III can be assessed by assuming that only one or two of the factors hold. The results are shown in Table 4.

**Table 3:** Increase in eNPV per investigational drug for phase III decentralized clinical trials (thousands 2020 USD): variation in assumed reduction in phase cycle time

Cycle time reduction (mos.)	eNPV delta	eNPV delta as percent of base eNPV (%)	ROI
1	$17,258	1.3	5.51x
2	$29,157	2.2	9.33x
3 (base analysis)	$41,158	3.2	13.17x
4	$53,263	4.1	17.04x
5	$65,471	5.0	20.94x
6	$77,785	6.0	24.88x

Costs and returns discounted to the start of phase III testing

*ROI* eNPV delta/Implementation cost

**Table 4:** Increase in eNPV per investigational drug for phase III decentralized clinical trials (thousands 2020 USD): variation in combination of screen failure, amendments and cycle time effects

Factor effects	eNPV delta	eNPV delta as percent of base eNPV (%)	ROI
Amendments only	− $2049	− 0.2	− 0.66×
Screen failure only	$5226	0.4	1.67×
Cycle time only	$33,323	2.6	10.66×
Screen failure plus amendments	$5460	0.4	1.75×
Amendments plus cycle time	$33,560	2.6	10.74×
Screen failure plus cycle time	$40,921	3.1	13.09×

Costs and returns discounted to the start of phase III testing

*ROI* eNPV delta/Implementation cost

Additional sensitivity analyses are shown in the Supplemental Data File. As is the case for phase II DCTs, the assumed number of trials in the phase has little impact on the value results. The increment in eNPV varies from $41,849,000 to $39,085,000 for two to six trials (Figure S4). Assumptions about the implementation cost have even less impact on the results for phase III than they do for phase II. A 25% lower implementation cost and a 25% higher implementation cost alters the increment in eNPV by only 1.4% relative to the base analysis (Figure S5). Finally, the impacts of a lower or higher assumed screen failure rate differential are modest. The increment in eNPV is 9.0% lower for a 5% screen failure rate differential and 9.8% higher for a 15% screen failure rate differential (Figure S6).

### Phase II Plus Phase III DCT Value

**Fig. 3:**
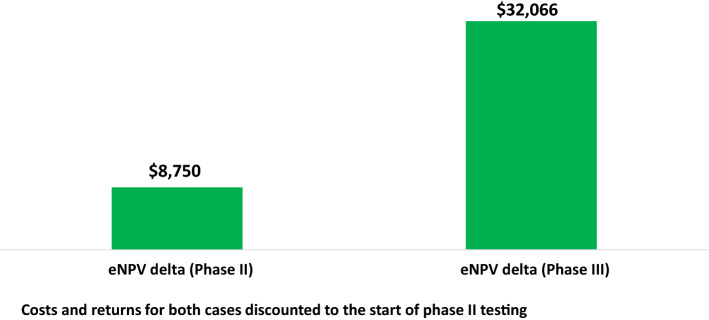
Increase in eNPV per Investigational Drug for Decentralized Clinical Trials (thousands 2020 USD): Phase II and Phase III Comparison

The increments in eNPV noted above are substantially higher for phase III compared to phase II ($41.2 million versus $8.6 million, respectively). However, that is, in a sense, not a fair comparison. The phase II cash flows are discounted back to the start of phase II, while the phase III cash flows are discounted back only to the start of phase III. A fair comparison would have the results expressed with discounting back to the same point in time. Figure 3 shows the comparison when the results for both cases are discounted back to the start of phase II testing ($32.1 million for phase III compared to $8.8 million for phase II). The primary reason for the much higher levels of and increments in value for phase III is that the likelihood that positive cash flows (sales) are realized is much higher for the set of drugs that have entered phase III testing compared to the set of drugs that have entered phase II testing. The likelihood of regulatory marketing approval is 56% for drugs that have entered phase III versus 19.9% for drugs that have entered phase II.

We have considered the net benefits of introducing DCT elements to clinical trials for phase II and phase III separately. However, we can also consider the implications if we apply DCT methods to both phase II and phase III trials. This is not a simple matter of adding the eNPV increment estimates that we have for the phases when considered separately, or even a simple average of the two. For the DCT case, we incorporate in our eNPV model the three factors identified as affecting eNPV, with the assumption that the factors will be applied to a portfolio of drugs that enter phase II testing for both phase II trials on all of those drugs and for phase III trials for the minority of these drugs that will progress to phase III (35.5%).

**Fig. 4:**
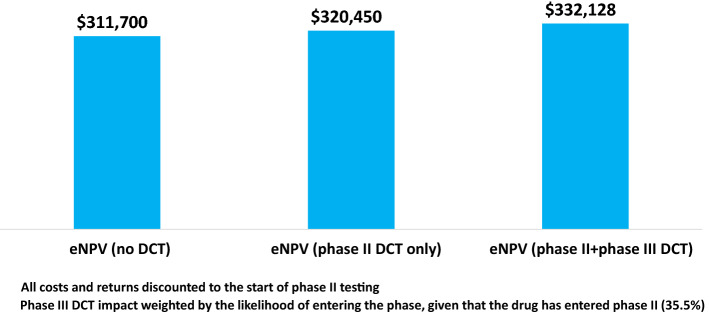
Increase in eNPV per Phase II Investigational Drug With Decentralized Clinical Trials for Both Phase II and Phase III (thousands 2020 USD)

Figure 4 shows the eNPV results for the status quo (non-DCTs), for DCTs for just phase II trials, and for DCT methods applied to both phase II and phase III trials. The increment in value in the phase II plus phase III DCT case versus no DCTs is $20,428,000. This eNPV increment is 6.6% of the base case (status quo) eNPV. Applying the weighted implementation cost noted above, the return on investment from applying DCT methods to both phase II and phase III trials is 6.81, or a ROI that is about seven times the investment cost.^2^

## Discussion

Throughout this assessment, as noted above, we have made conservative assumptions for our financial modeling. The results of this analysis show substantial net financial returns on DCT investments made by sponsor companies to support clinical trial execution. The financial gains in this initial model are driven by shorter cycle times and lower mean clinical trial costs associated with DCT deployments. Relative performance and cost differences are likely the result of increases in study volunteer willingness to participate due to improvements in clinical trial access and convenience and in the lower mean number of protocol amendments typically associated with efforts to ameliorate the difficulties in enrolling study volunteers.

The results of this ROI assessment also do not take into account efficiencies gained over time as a result of greater familiarity with virtual and remote technologies. The upfront costs and time associated with personnel training, for example, are expected to decrease as sponsors, CROs, and investigative sites gain experience with a given DCT solution. The results also do not capture the potential efficiencies gained over time as a result of more automated data collection activity, which remains to be evaluated.

There are no doubt challenges associated with DCT use in adaptive designs and trials relying on interim analyses but the vast majority of deployments and the general experience with DCTs has not yet reached this more advanced and nuanced stage. To our knowledge, there are no data, nor are there anecdotal reports or published observations at this time that we can cite or include in the narrative. We acknowledge that these are challenges but understand that the DCT adoption experience is still in its early stages and that documentation is nascent.

The impacts on sponsor net financial benefits can be made more precise with additional data of the type we examined for this study. Future research will focus on gathering relevant information on aspects of clinical trial operations for which we currently do not have adequate data (e.g., dose adherence, automated communication and data collection, patient population generalizability, and data monitoring efficiencies).

We anticipate that the impacts of DCT methods will vary by drug therapeutic class or other drug or patient population characteristics. In future research, it would be useful to gather the data necessary to examine the impact of DCT methods on oncology development, currently the largest area of new drug development and where returns may differ from those for other therapeutic areas, and for drugs to treat rare diseases where the patient population is small and visits to central sites may therefore be very costly and burdensome.

Tufts CSDD is also planning to conduct a longitudinal study with data on actual sponsor company experience with specific DCT approaches and technologies. This will facilitate a much larger and broader analysis by TA, participant demographics, trial phase, trial complexity and more. With expanded data we may find that there are settings in which the benefits of DCTs are relatively low because the opportunities to employ DCTs in those settings are limited. In some the costs of implementing DCTs could exceed the benefits. The analysis here applies industry-wide trial averages under the assumption that the technology would be applied across the board. However, if, given a more granular analysis, the technology is applied judiciously then we would expect the net benefit of DCTs per investigational drug to be higher than what we found when one assumes that the technology is applied uniformly. In that sense, our current results are conservative. Our aim is to harness the knowledge gained in this and subsequent analyses to optimize the use of DCTs.

We note that the net benefits measured in this study are those that accrue to drug developers. The social benefits of employing DCT methods would include these benefits, but also include benefits enjoyed by trial participants. These can include improved access and convenience, and perhaps greater participant satisfaction from remote monitoring capabilities that are not otherwise available without extensive clinical support time. It would therefore be useful to have the data necessary to examine these aspects of virtual and remote approaches and technologies to quantify societal benefits fully.

Finally, we note the potential impact of DCTs on trial sites. We do not have specific information about site-related workloads and site costs, but we may gather such detailed information in our follow-on work. However, our experience is that we have not seen evidence of additional costs associated with managing tests or treatments. DCTs as a whole should not equate to additional tests or treatments but rather a use of digital approaches in overall trial conduct. The only area we have seen a significant impact on site time has been around training. The data we have seen is that there has been a significant increase in the training burden as a result of the addition of multiple tech streams and this burden was captured by *SCRS* [21] in their 2021 annual survey of sites. Their effort quantified the impact as an additional 17.5 h per study, per site. This burden seems unsustainable and our expectations are that some of this effort can be tied to change management and will decrease over time. We presume that any extra burden, if it persisted, would be reflected in the contract value data.

## Conclusions

We have examined the impact that remote and virtual methods and technologies to collect study data, collectively known as DCTs, have on drug sponsor finances. The evaluations are conducted in the context of a risk-adjusted discounted cash flow analysis, or eNPV model. The analyses show substantial net benefit to drug developers. The increase in eNPV for a portfolio of phase II investigational drugs was $8.8 million per drug, with a five-fold ROI. When considering a portfolio of phase III drugs, employing DCT methods increased value by $41 million per drug, with a 13-fold ROI. If we assume that DCT methods are applied to both phase II and phase III trials the increase in value is $20 million per drug that enters phase II, with a seven-fold ROI.

The factors that impact development finances positively that we examined are reductions in phase durations, decreases in screen failure rates, and fewer substantive protocol amendments.

In this analysis, the benefits to employing DCT methods are driven overwhelmingly by reductions in phase cycle times. Fewer protocol amendments, while contributing to sponsor financial benefits, were not sufficient on their own to create a net financial benefit to DCTs considering the current costs of implementing DCT programs. The same can be noted for screen failure rate differentials. However, reductions in screen failure rates in conjunction with fewer substantial amendments have a modest positive impact on increases in value for DCTs.

### Limitations

We did not focus on specific diseases or qualify inclusion based upon disease incidence or prevalence. The Medable data used in this study is for trials representing a variety of therapeutic classes and are consciously not overweighted in one drug type or therapeutic area (TA) but balanced to be fairly representative of the industry. Studies included also represent those farther along in enrollment as this was evidence of successful implementation compared with those not yet actively enrolling. It is possible that for rare diseases, the eNPV values and ROIs may be significantly higher as DCT methods may be critical to rapid enrollment when cases are geographically sparse.

While specific assumptions have been made regarding the value drivers, we have conducted and documented sensitivity analyses to assess the relative impact of these assumptions. It would still be beneficial to conduct an analysis of a larger dataset to evaluate impacts by TAs, disease incidence, and variation in the complexity of trials. This will help illustrate the relative impacts that DCTs could make where the value might be greatest in relation to assumed costs.

## Supplementary Information

Below is the link to the electronic supplementary material.

Supplementary file1 (DOCX 45 kb)

## Footnotes

1 If deploying DCT approaches and technologies decrease clinical trial durations (as we find below), then it is possible that effective patent lifetime increases for some drugs. However, legislated restoration of patent life lost during clinical development is restricted in several ways by the legislation and so may not have any effect for many drugs. Furthermore, the extension of patent life, when it does occur, happens at the end of the patent protection period. The value of the extra time without generic competition occurs far in the future relative to the start of phase II testing, and so discounting would substantially reduce the value of the extended protection. To be conservative, we assume no extension of patent protection in the model.

2 Although the results will not be exact, one can get close to the increment in eNPV measured here more simply by weighting the results we have for the phases individually. To illustrate, suppose that we have 1,000 investigational drugs that are going to enter phase II testing. Given the phase II transition rate that we have been using, only 355 of the drugs in this portfolio will proceed to phase III testing. We found the increments in eNPV from DCTs to be $8,750,00 for phase II and $32,066,000 for phase III. Using these numbers, the total increment in value from applying DCT methods to both phases would be (1000 x $8,750,000) + (355 x $32,066,000) = $20,133,430,000, or $20,133,430 per phase II investigational drug. This is very close to the value we get from applying the model directly as described in the text. The ROI in this case is also therefore very close at 6.71.
