# Supplementary material for: Assessing the Financial Value of Decentralized Clinical Trials
Source: Ther Innov Regul Sci. 2022 Sep 14;57(2):209–19. doi: 10.1007/s43441-022-00454-5 (PMC9473466; doi:10.1007/s43441-022-00454-5)
Supplement: Supplementary file 1 — Supplementary file1 (DOCX 45 kb) [file 43441_2022_454_MOESM1_ESM.docx]

**SUPPLEMENTAL DATA FILE**

**ASSESSING THE FINANCIAL VALUE OF DECENTRALIZED CLINICAL TRIALS**

**Joseph A. DiMasi,^1^ Zachary Smith,^1^ Ingrid Oakley-Girvan,^2^ Andrew Mackinnon,^2^ Mary Costello,^2^ Pamela Tenaerts,^2^ Kenneth A. Getz^1^**

**^1^ Tufts Center for the Study of Drug Development, Tufts University**

**^2^ Medable, Inc**

**I. TECHNOLOGY DEFINITIONS AND PLATFORMS**

**CTTI Decentralized Clinical Trial definition:**

Decentralized clinical trials (DCTs) are those in which some or all study assessments or visits are conducted at locations other than the investigator site via any or all of the following DCT elements: tele-visits; mobile or local healthcare providers, including local labs and imaging centers; and home delivery of investigational products. Decentralized clinical trials can be completely remote or partially decentralized with hybrid approaches. Hybrid trials are those that require some visits to be conducted on site, while other visits or assessments can be performed at a participant’s home or within their local care community. Fully remote trials have no required site visits.

Source: <https://ctti-clinicaltrials.org/wp-content/uploads/2022/04/CTTI-Digital-Health-Trials-Planning-Decentralized-Trials-Recs.pdf>

**Medable DCT Technology:**

Medable’s mission is to get effective therapies to patients faster by transforming clinical drug development. The company’s digital platform streamlines design, recruitment, retention, and data quality for decentralized trials, accelerating the science behind clinical trials. Medable connects patients, sites, and clinical trial teams to improve patient access, experience, and outcomes. Medable is a privately held, venture-backed company headquartered in Palo Alto, California.

The Medable technology modules can be described as follows:

 (1) eConsent

Medable eConsent incorporates multimedia and supplementary sections that can aid understanding of the consent to improve comprehension. The consent can be available as pre-read/review by patients and then actively discussed with the patient and clinician to address questions and assure comprehension prior to executing signatures. eConsent is available on-site in a face-to-face meeting and/or within a unified remote workflow incorporating an on-platform televisit. This allows for face-to-face interaction and discussion to continue and provides the time/date/participant IDs of the televisit record to be evidenced alongside the ICF signature execution data. The platform allows a study participant to download their executed documents at any time as well as have them accessible for recall through the duration of the study. Any reconsents follow the same process and the patient has access to all versions executed.

 (2) eCOA (includes ePRO)

The Medable applications include ePRO/eDiary for patients as well as eCOA/eSource for Clinicians. Medable offers configurable tasks for the collection of demographics, medical history and concomitant medications as required per protocol design. Medable also offers image capture (medications, OTC medications etc.), bar code scans (kit receipt) and direct integration to bluetooth-connected devices and wearables for data upload, or via cloud-to-cloud integrations. Data directly connected into patient app is automatically viewable to stakeholders (with permissions) on platform as well as site staff with the ePRO, diaries and other patient reported data. Medable also offers the ability for patient reported adverse event reporting workflow to sites, which can be further integrated to an EDC or direct to safety team.

 (3) BYOD

The Medable Patient Application operates as a portal that enables the patient to access all activities related to the study via a single source. The patient can access the application via provisioned devices or BYOD (iOS, Android, and Web) which offers full flexibility to engage patients through the modality with which they feel most comfortable and familiar. The platform is designed to facilitate patient-centered research and direct patient data capture, optimizing the patient experience and allowing each patient a consolidated view of the study, collected data, prospective and upcoming activities as well as options to engage with PI/site team, such as TeleVisits. The patient facing features include: eConsent, self reported screening/eligibility, ePRO, patient diaries (static and dynamic formats), integrated devices, reminders/notifications, telemedicine, study resources, and image capture. Medable also offers the ability to integrate with other systems, such as patient reimbursement solutions, to unify the experience for patients.

 (4) TeleVisits

Medable's on-platform TeleVisit product is available in the Patient and Clinician Applications via the Web, iOS, and Android. No installation is required when utilizing the web application and the native mobile applications are available via iOS and Android which through the Apple and GooglePlay stores in a BYOD setting or fully configured in a provisioned model including Mobile Device Management.

**II. eNPV MODEL PARAMETERS**

**Table S1. Base case parameter values and assumptions per investigational drug for decentralized clinical trial eNPV model for phase II interventions**

| **Development Characteristics^a^** | | | | | |
| --- | --- | --- | --- | --- | --- |
| ***R&D costs (millions 2020 USD)*** |  | ***Development time (mos.)*** |  | ***Development risk*** |  |
| Phase II | $65.4 | Phase II to phase III | 30 | Probability of phase III | 35.5% |
| Phase III | $285.2 | Phase III to registration | 31 | Probability of registration | 22.0% |
| Post-approval R&D | 25% of total costs | Registration to approval | 16 | Probability of approval | 19.9% |
| **Commercialization Characteristics^b^** | | | | | |
| ***Marketing costs (% of sales)*** |  | ***Operational costs (% of sales)*** |  | ***Finance and Government*** |  |
| Launch costs (pre-approval) | 5% of peak sales (1 year) | COGS | 30% | Cost of Capital | 10.5% |
| Launch costs (post-approval) | 5% of peak sales (1 year) | Medical Affairs | 5% | Effective tax rate | 18.0% |
| Steady state | 20% | Other | 2% |  |  |
| ***Sales Curve*** |  |  | | | |
| Peak year sales (millions 2020 USD)^c^ | $1,852 |  | | | |
| Years to peak sales^c^ | 10 |  | | | |
| Exclusivity period (yrs.) | 11 |  | | | |

^a^ Source: DiMasi JA, et al. *J Health Econ* 2016;47:20-33

^b^ Source: industry standards

^c^ Source of data: actual and consensus future sales forecasts from *Cortellis* and *Adis Insight* pipeline databases

**Table S2. Base case parameter values and assumptions per investigational drug for decentralized clinical trial eNPV model for phase III interventions**

| **Development Characteristics^a^** | | | | | |
| --- | --- | --- | --- | --- | --- |
| ***R&D costs (millions 2020 USD)*** |  | ***Development time (mos.)*** |  | ***Development risk*** |  |
| Phase III | $285.2 | Phase III to registration | 31 | Probability of registration | 62.0% |
| Post-approval R&D | 25% of total costs | Registration to approval | 16 | Probability of approval | 56.0% |
| **Commercialization Characteristics^b^** | | | | | |
| ***Marketing costs (% of sales)*** |  | ***Operational costs (% of sales)*** |  | ***Finance and Government*** |  |
| Launch costs (pre-approval) | 5% of peak sales (1 year) | COGS | 30% | Cost of Capital | 10.5% |
| Launch costs (post-approval) | 5% of peak sales (1 year) | Medical Affairs | 5% | Effective tax rate | 18.0% |
| Steady state | 20% | Other | 2% |  |  |
| ***Sales Curve*** |  |  | | | |
| Peak year sales (millions 2020 USD)^c^ | $1,852 |  | | | |
| Years to peak sales^c^ | 10 |  | | | |
| Exclusivity period (yrs.) | 11 |  | | | |

^a^ Source: DiMasi JA, et al. *J Health Econ* 2016;47:20-33

^b^ Source: industry standards

^c^ Source of data: actual and consensus future sales forecasts from *Cortellis* and *Adis Insight* pipeline databases

**III. THERAPEUTIC CLASS DISTRIBUTIONS FOR TUFTS CSDD AND MEDABLE DATASETS**

**Table S3. Therapeutic Class Distributions for Tufts CSDD Benchmark Protocol and Medable Databases for Phase II and Phase III Clinical Trials***

| **Class** | **Medable** | **Tufts CSDD** |
| --- | --- | --- |
| Oncology | 27.1% (16) | 33.1% (53) |
| Endocrinology/Metabolic | 10.2% (6) | 5.0% (8) |
| Infectious Disease | 6.8% (4) | 5.6% (9) |
| Cardiovascular | 5.1% (3) | 6.9% (11) |
| Neurology | 5.1% (3) | 13.1% (21) |
| Immunology | 3.4% (2) | 12.5% (20) |
| Other | 42.4% (25) | 23.8% (38) |

^*^ Classes with at least 5% of the sample

**IV. SENSITIVITY ANALYSES**
